# The risk of elevated prolactin levels in pediatric patients exposed to antipsychotics for the treatment of schizophrenia and schizophrenia spectrum disorders: protocol for a systematic review and meta-analysis

**DOI:** 10.1186/2046-4053-3-116

**Published:** 2014-10-13

**Authors:** Eric Druyts, Shawn Eapen, Ping Wu, Kristian Thorlund

**Affiliations:** 1 Faculty of Health Sciences, University of Ottawa, 25 University Private, Ottawa, Ontario K1N 6N5, Canada; 2 Department of Clinical Epidemiology and Biostatistics, McMaster University, 1280 Main Street West, Hamilton, Ontario L8S 4K1, Canada; 3 Stanford Prevention Research Center, Stanford University, 291 Campus Drive, Stanford, CA 94305-5101, USA

**Keywords:** Prolactin, Pediatric, Children, Adolescents, Antipsychotics, Schizophrenia, Systematic review, Meta-analysis

## Abstract

**Background:**

Antipsychotic medications, particularly second-generation antipsychotics, are increasingly being used to alleviate the symptoms of schizophrenia and other severe mental disorders in the pediatric population. While evidence-based approaches examining efficacy and safety outcomes have been reported, no review has evaluated prolactin-based adverse events for antipsychotic treatments in schizophrenia and schizophrenia spectrum disorders.

**Methods/design:**

Searches involving MEDLINE, EMBASE, CENTRAL, PsycINFO, and clinical trial registries (ClinicalTrials.gov, Drug Industry Document Archive [DIDA], International Clinical Trials Registry Platform [ICTRP]) will be used to identify relevant studies. Two reviewers will independently screen abstracts and relevant full-text articles of the papers identified by the initial search according to the prospectively defined eligibility criteria. Data extraction will be conducted in duplicate independently. Pairwise random effects meta-analyses and network meta-analyses will be conducted on individual drug and class effects where appropriate.

**Discussion:**

This systematic review will evaluate prolactin-based adverse events of first- and second-generation antipsychotics in the pediatric population with schizophrenia and schizophrenia spectrum disorders. It will also seek to strengthen the evidence base of the safety of antipsychotics by incorporating both randomized controlled trials and observational studies.

**Systematic review registration:**

PROSPERO CRD42014009506

## Background

Antipsychotics are a cornerstone in the treatment of patients with severe mental disorders, including schizophrenia and other schizophrenia spectrum disorders. The pediatric use of antipsychotics has substantially increased since the introduction of second-generation antipsychotics (SGAs) (also known as ‘atypical’ antipsychotics), despite limited evidence supporting their efficacy and safety in this population [1,2]. In Canada, the prevalence of antipsychotic use in those aged 18 years or younger increased from 1.9 per 1,000 in 1999 to 7.4 per 1,000 in 2008, with more than 70% of these prescriptions being written by general practitioners [3], despite the lack of approval for pediatric use by Health Canada. As such, there is a need for post-marketing surveillance activities of antipsychotic use in children [4].

Children may be more susceptible to antipsychotic adverse effects than adults largely due to developmental physiological changes that occur in childhood. However, few studies have systematically monitored the safety of antipsychotics in pediatric populations [5]. SGAs have long been considered a safer alternative to first-generation antipsychotics (FGAs) (also known as ‘typical’ antipsychotics) due to their lower tendency to induce neurological effects. However, other equally problematic adverse effects have been associated with SGAs, leading experts to have concerns about the proper utilization of these agents, especially in pediatric patients [6,7].

SGAs elevate serum prolactin via dopamine antagonism [8-10]. Studies have shown that risperidone, for example, may elevate prolactin to a greater extent and more frequently than other SGAs in pediatric and adult populations [11-14]. Prolonged and substantial elevation of prolactin is associated with a number of adverse effects. Direct effects of elevated prolactin on breast tissue may lead to galactorrhea in women and gynecomastia in men [15]. Other effects include amenorrhea, sexual dysfunction, and osteoporosis in women and erectile dysfunction and impaired spermatogenesis in men [16]. Interaction of antipsychotic drugs with dopamine receptors is thought to be important for their mechanisms of action, leading to both therapeutic and adverse effects [17-19]. Good evidence supports the existence of both metabolic and neurological adverse effects in children treated with these medications [20].

In 2011, a group known as the Canadian Alliance for Monitoring Effectiveness and Safety of Antipsychotics in Children (CAMESA) published management recommendations for metabolic complications associated with SGA use in pediatric patients [21]. They discussed that elevated levels of prolactin may be associated with adverse events such as gynecomastia, galactorrhea, infertility, menstrual irregularities, oligomenorrhea, amenorrhea, sexual dysfunction, decreased libido, acne, and hirsutism in females [21]. However, hyperprolactinemia may be asymptomatic, particularly in a pre-pubescent population. The authors acknowledge that the evidence base of clinical consequences of elevated prolactin levels is sparse, and therefore, their recommendations on how to manage patients with hyperprolactinemia are based on expert consensus opinion [21].

Although risperidone is approved for use in pediatric patients in the United States, it is not approved for use in this population in Canada. Despite this, there is considerable off-label use of risperidone in Canadian pediatric patients [22]. To date, no systematic reviews have comparatively examined the prolactin-related adverse event profiles of both the FGAs and SGAs in schizophrenia and schizophrenia spectrum disorder pediatric patients. The hypothesis of a correlation between the dopamine receptor affinity of these agents and an increase in the risk of prolactin-related adverse events has also not been tested. With the increased off-label use of these medications, it is important that we further examine whether one or more of these drugs may produce unreasonable risks to pediatric patients. The information generated by the proposed systematic review can help to determine the necessary follow-up steps to minimize risks associated with the elevated prolactin levels linked to certain antipsychotics.

## Methods/design

### Objective

The objective of the proposed project is to examine the risk of prolactin-related adverse events associated with the use of antipsychotic medications for the treatment of schizophrenia and schizophrenia spectrum disorders in pediatric patients. We propose the use of meta-analytic techniques to compare the frequencies of prolactin-related adverse events associated with each of the antipsychotics. We will conduct our analyses using two approaches: (1) considering each drug independently and (2) considering all drugs within a given dopamine receptor affinity class as exhibiting comparable treatment effects.

### Eligibility criteria

Eligibility criteria for study selection are described according to the population-intervention-comparators-outcomes-study design (PICOS) structure. Specifically, published studies comparing relevant interventions in human subjects will be eligible if the following criteria are met:

#### Population

Pediatric patients (children and adolescents aged 5–18 years) diagnosed with schizophrenia and schizophrenia spectrum disorders will be considered.

Note that antipsychotic monotherapy is acknowledged as the treatment of choice for patients with schizophrenia [23]. Given the propensity for polypharmacy in other conditions, such as bipolar disorder, schizophrenia is the key focus in an attempt to ensure the true effect of antipsychotics on prolactin-related adverse events is examined.

#### Interventions/comparisons

Commonly used FGAs and SGAs will be considered (see Table 1). Placebo and no treatment will be considered to be relevant comparator treatments, where applicable.

**Table 1:** Interventions of interest

**First-generation antipsychotics (FGAs)**	**Second-generation antipsychotics (SGAs)**
Chlorpromazine (Thorazine)	Aripiprazole (Abilify)
Fluphenazine (Prolixin)	Asenapine (Saphris)
Haloperidol (Haldol)	Clozapine (Clozaril)
Loxapine (Loxitane)	Iloperidone (Fanapt)
Molindone (Moban)	Olanzapine (Zyprexa)
Perphenazine (Trilafon)	Paliperidone (Invega, Invega Sustenna)
Thioridazine (Mellaril)	Risperidone (Risperdal)
Thiothixene (Navane)	Quetiapine (Seroquel)
Trifluoperazine (Stelazine)	Ziprasidone (Geodon)

#### Outcomes

Outcomes will include prolactin-related adverse events: gynecomastia, galactorrhea, sexual dysfunction (impotence/decreased libido), menstrual irregularities (amenorrhea/dysmenorrhea), change in prolactin levels, and any other prolactin-related adverse event reported in the included studies.

#### Study design

Both clinical trials and prospective observational studies will be included as data may be limited if we were to restrict to one study design. Data from both designs may be meta-analyzed together if they report common information; however, based on preliminary scoping and readings, we anticipate conducting a variety of analyses. The study lengths of follow-up and reporting times of outcomes will inform the choice of time duration upon which analyses will be based. Multiple time points are feasible, and it is expected that some studies will have limited follow-up.

### Literature search and study selection

We have developed electronic search strategies with the assistance of a medical librarian at the University of Ottawa for the following databases: MEDLINE, EMBASE, CENTRAL, and PsycINFO. Our search strategies consist of terms related to the specific interventions of interest and a pediatric patient population (see the Appendix for the MEDLINE/EMBASE search strategy). No schizophrenia terms are used in the search strategy in order to be inclusive of all schizophrenia and schizophrenia spectrum disorders. With changes of the Diagnostic and Statistical Manual of Mental Disorders (DSM) over time, the definitions and diagnostic criteria of schizophrenia and schizophrenia spectrum disorders have evolved [24]. The bibliographies of all identified relevant studies will be used to perform a recursive search of the literature.

We will also conduct a comprehensive gray literature search. This will include clinical trial registries (ClinicalTrials.gov and International Clinical Trials Registry Platform [ICTRP]), drug industry documentation (Drug Industry Document Archive [DIDA]), and publically available clinical study reports (CSRs).

Articles will be assessed according to the prospectively defined eligibility criteria by two independent reviewers using pre-designed eligibility forms. Abstracts of papers identified by the initial search will be evaluated by the lead reviewer for appropriateness to the study question, and all potentially relevant papers will be obtained and evaluated in detail.

### Data extraction

After relevant studies have been identified, we will meet as a group to ensure eligibility. Data extraction will be conducted in duplicate independently. Among included studies, we will extract data on the study design and inclusion criteria, intervention dosage, pill count, control (if appropriate), features of patient status (e.g., age, gender, race, co-morbidities, functional status indicators, recovery rates), duration of follow-up, co-interventions, and outcomes of interest. The outcomes of interest (e.g., gynecomastia, galactorrhea, sexual dysfunction, menstrual irregularities, and change in prolactin levels) will be extracted individually rather than as a composite of prolactin-related adverse events. We are aware that some outcomes may be rarely reported. The non-reporting of a particular outcome will not constitute the reporting of a zero count. Trials that do not report a particular outcome will not be included in the subsequent analyses.

### Assessment of risk of bias

We will assess the validity of included trials using the Cochrane risk of bias instrument. This instrument evaluates six key domains: sequence generation; allocation concealment; blinding of participants, personnel, and outcome assessors; incomplete outcome data; selective outcome reporting; and other sources of bias [25]. We will modify response options for assessment of blinding by substituting ‘probably yes’ and ‘probably no’ for ‘unclear,’ as we have recently demonstrated that this approach can enhance the assessment of blinding in randomized trials [26].

We will employ the Grading of Recommendations Assessment, Development and Evaluation (GRADE) system for rating the overall quality of evidence [27]. For each outcome, randomized trials begin as high-quality evidence but may be rated down by one or more of five categories of limitations: (1) risk of bias, (2) consistency, (3) directness, (4) imprecision, and (5) reporting bias. The quality of evidence for each main outcome can be determined after considering each of these elements and can be categorized as either high (we are very confident that the true effect lies close to that of the estimate of the effect), moderate (we are moderately confident in the effect estimate: the true effect is likely to be close to the estimate of the effect, but there is a possibility that it is substantially different), low (our confidence in the effect estimate is limited: the true effect may be substantially different from the estimate of the effect), or very low (we have very little confidence in the effect estimate: the true effect is likely to be substantially different from the estimate of effect) [27]. Furthermore, we will make use of the recent paper by Salanti et al. to determine the quality of evidence from a network meta-analysis [28]. We will include (1) the key role of indirect comparisons, (2) the contributions of each piece of direct evidence to the network meta-analysis estimates of effect size, (3) the importance of the transitivity assumption to the validity of network meta-analysis, and (4) the possibility of disagreement between direct evidence and indirect evidence [28].

For included observational studies, we will assess the validity of the individual studies using the Newcastle-Ottawa Scale (NOS) [29]. This instrument is used to assess selection of patients, comparability of cohorts (or cases and controls), and adequacy of outcomes/exposures [29].

### Evidence synthesis

We will conduct our analyses using two different approaches: (1) considering each drug as independent and (2) considering all drugs within a given dopamine receptor affinity class as exhibiting comparable treatment effects. The first approach will allow us to examine if specific drugs exhibit a certain effect over others. Although this is a naïve approach that will ignore the fact that drugs of a similar classification are likely to exhibit similar magnitudes of treatment effect, there is evidence that certain antipsychotic drugs of the same class have differing adverse event profiles. The second approach will allow us to examine a critical pharmacokinetic property, dopamine receptor affinity, which is highly linked to the regulation of prolactin release.

#### Statistical model for clinical trial data

Pairwise meta-analysis and, where appropriate, network meta-analysis will be conducted for each prolactin-related adverse event outcome. Network meta-analyses gain their strength from utilizing evidence from all available randomized control trials (RCTs) that have compared any treatment of interest to a control intervention (e.g., placebo) or head-to-head with another treatment of interest [30,31]. Network meta-analyses thus allow for strong inferences about comparative effectiveness between all interventions, even when interventions have never or rarely been compared head-to-head [31].

Placebo/no therapy will be used as the reference therapy. Posterior densities for unknown parameters will be estimated using Markov chain Monte Carlo (MCMC) methods. Non-informative or vague prior distributions will be used throughout, allowing the data to drive the final inferences.

#### Addressing heterogeneity

Primary analyses will be unadjusted analyses, and then additional analyses to assess heterogeneity will be pursued [32]. A feasibility assessment of the distribution of study and patient characteristics that may affect treatment effects across direct comparisons of the evidence networks will be conducted [33]. We will first create a list of potential treatment effect modifiers for the interventions of interest based on prior knowledge or subgroup results of individual studies before comparing results between studies. Next, the distribution of study and patient characteristics that are determined to be likely effect modifiers will be compared across studies to identify any potential imbalances between different types of direct comparisons. Where possible, sensitivity analyses will be undertaken if there are important variations in the definitions of the outcomes across studies. Furthermore, where feasible, meta-regression will be incorporated into the models to test and (potentially) control for effect modification.

#### Secondary analysis using observational data

To incorporate observational data, a two-stage approach will be applied. First, all comparisons informed by observational data will be meta-analyzed using pairwise random effects meta-analysis. From each of these pairwise meta-analyses, an approximate normal distribution of the mean log odds ratio will be generated with some mean and variance estimates. Second, the generated approximate distribution will be used as a prior distribution for the particular comparison (i.e., log odds ratio) in the Bayesian network meta-analysis model described above. As sensitivity analyses, less information versions using the same means but twice and four times the estimated variances (i.e., half and a quarter of the precision) will also be carried out.

#### Reporting of findings

Full graphical and numeric presentations of findings along with a layperson’s summary will be provided to convey the results of our work. This will include the following: network diagrams showing the availability of evidence for all possible treatment comparisons; summary odds ratios and 95% credible intervals for all pairwise comparisons, both in tabular form and in forest plots; and estimates of probabilities that each therapy is deemed ‘best’ for each outcome along with associated average rankings of efficacy and safety; these will be described using approaches recommended by Salanti et al. [34].

## Discussion

This proposed review will add to the literature in several ways. Many existing reviews of pediatric populations focus solely on the efficacy and tolerability outcomes [35,36]. To our knowledge, prolactin-related adverse events have only briefly been assessed in prior meta-analyses of antipsychotic drugs. Seida et al. completed a comparative effectiveness review on antipsychotic use in a combined pediatric and young adult population. While the focus was efficacy outcomes, prolactin-related and sexual side effects (amenorrhea, oligomenorrhea, erectile dysfunction, decreased libido, hirsutism, breast symptoms, galactorrhea, prolactin levels) were combined and analyzed together [37]. Furthermore, only efficacy outcomes were stratified by indication; safety outcomes were analyzed by drug class for all indications. Other studies report changes in prolactin levels but not specifically in schizophrenia or schizophrenia spectrum disorders [20,38,39]. Therefore, this proposed review allows us to (1) inform treatment guidelines for pediatric schizophrenia and schizophrenia spectrum disorder and (2) provide evidence for specific prolactin-related adverse events.

Furthermore, the comparative risks between newer and older antipsychotics have not been well defined as of yet. A systematic approach to quantifying the risks for both groups will provide the safety perspective required for clinicians to make evidence-based treatment decisions.

There are a few potential limitations of this planned review. Clinical expertise and preliminary review of some of the relevant studies for this research shows that in many cases, while patients are randomized to one active antipsychotic medication to address the study’s research question, additional concomitant medications for the treatment of their disorder may be prescribed to patients at physicians’ discretion. While these additional treatments may have implications for additional effects seen, outcome data for those remaining strictly on the prescribed treatments may not be available. This will be a limiting factor of the purity of some of the included trial data. We will address this issue by exploring the associated frequencies and reasons for changes.

The largest studies available will likely be retrospective observational studies and, as such, may be prone to various degrees of residual confounding due to the lack of complete information (e.g., severity of patients’ condition, timing of drug use, co-morbidities). While it will be feasible to capture and comment on the extent of information collected and adjusted for, the impact of any missing information will not be evaluable.

As the majority of existing evidence on antipsychotic medications is from adult populations, the results of this systematic review and meta-analyses will be of interest to key stakeholders, policy makers, researchers, and clinicians working in pediatric mental health care. With the growing need for age-specific research in schizophrenia and schizophrenia spectrum disorders, this review seeks to highlight prolactin-related adverse event profiles in the pediatric population and to strengthen the evidence base of the safety of antipsychotics by incorporating both randomized controlled trials and observational studies.

## Appendix

### Medline/EMBASE search strategy

1. exp Infant/

2. (Infant* or infancy or Newborn* or Baby* or Babies or Neonat* or Preterm*).mp.

3. exp Child/

4. (Child* or Schoolchild* or School age* or Preschool* or Kid or kids or Toddler*).mp.

5. exp Adolescent/

6. Adoles*.ti,ab.

7. (Teen* or Boy* or Girl*).mp.

8. exp Minors/

9. minors*.mp.

10. exp Puberty/

11. (Pubert* or Pubescen* or Prepubescen*).mp.

12. exp Pediatrics/

13. (Pediatric* or Paediatric* or Peadiatric*).mp.

14. exp Schools/

15. (Nursery school* or Kindergar* or Primary school* or Secondary school* or Elementary school* or High school* or Highschool*).mp.

16. or/1-15

17. exp Adolescent/ and exp Adult/

18. or/1-4,6-15

19. 17 not 18

20. 16 not 19

21. chlorpromazine/

22. (chlorpromazine or Thorazine or Largactil or Megaphen).ti,ab,kw.

23. fluphenazine/

24. (fluphenazine or Prolixin).ti,ab,kw.

25. haloperidol/

26. (haloperidol or Haldol or Sernace or Haldol*).ti,ab,kw.

27. loxapine/

28. (loxapine or loxitine or Loxapac or Loxitane or Adasuve).ti,ab,kw.

29. molindone/

30. (molindone or Moban or Moban*).ti,ab,kw.

31. perphenazine/

32. (perphenazine or Trilafon or Trilafon*).ti,ab,kw.

33. thioridazine/

34. (thioridazine or Mellaril or Melleril or Sonapax or Thioril).ti,ab,kw.

35. thiothixene/

36. (thiothixene or tiotixene or Navane).ti,ab,kw.

37. trifluoperazine/

38. (trifluoperazine or Eskazinyl or Eskazine or Jatroneural or Modalina or Stelazine or Terfluzine or Trifluoperaz or Triftazin).ti,ab,kw.

39. aripiprazole/

40. (aripiprazole or Abilify or Aripiprex).ti,ab,kw.

41. asenapine/

42. (asenapine or Saphris or Sycrest).ti,ab,kw.

43. clozapine/

44. (clozapine or Clozaril or Clozaril*).ti,ab,kw.

45. iloperidone/

46. (iloperidone or Fanapt or Fanapta or Zomaril or Fanapt*).ti,ab,kw.

47. olanzapine/

48. (olanzapine or Lanzek or Zypadhera or Oleanz or Zyprexa).ti,ab,kw.

49. paliperidone/

50. (paliperidone or Invega or Invega*).ti,ab,kw.

51. risperidone/

52. (risperidone or Risperdal or Risperdal Consta or Risperdal M-Tab or Risperdal Quicklets or Risperdal*).ti,ab,kw.

53. quetiapine/

54. (quetiapine or Seroquel or Seroquel*).ti,ab,kw.

55. ziprasidone/

56. (ziprasidone or Geodon or Zeldox or Zipwell or Geodon*).ti,ab,kw.

57. or/21-56

58. 20 and 57

59. limit 58 to human [Limit not valid in CCTR; records were retained]

60. limit 59 to english language

61. remove duplicates from 60

## Competing interests

Eric Druyts and Ping Wu are contractors to Redwood Outcomes. Kristian Thorlund is a founding partner of Redwood Outcomes and has consulted Boehringer Ingelheim, Merck, Pfizer, Novartis, Janssen, Roche, Novo Nordisk, UCB, and Gilead on systematic reviews and meta-analyses. Shawn Eapen declares no competing interests.

## Authors’ contributions

ED, SE, PW, and KT contributed equally to the development of this protocol. They conceived of the study, participated in its design and coordination, and helped to draft the manuscript. All authors read and approved the final manuscript.
